# Role of Open AI (Artificial Intelligence)–Innovations in Health Behavior Change Interventions

**DOI:** 10.3390/healthcare11202710

**Published:** 2023-10-11

**Authors:** Amar Kanekar

**Affiliations:** School of Counseling, Human Performance and Rehabilitation, University of Arkansas at Little Rock, 2801 South University Avenue, Little Rock, AR 72204, USA; axkanekar@ualr.edu

The call for articles for the special section entitled ‘Innovations in Health Behavior Change’ is currently open and is gaining interest from editors and authors worldwide. Although this call seeks innovations in health behavior theories, models and their applications, it also alludes to innovations in use of technology that change how health-related risky behaviors are assessed and prevented. Thus far, the articles published in this Special Issue have demonstrated diverse applications of technology such as the use of virtual communities, internet-based imagery applications, mobile health interventions and innovative tools in pediatric dentistry practice. With recent developments in the field of Open AI and its applications in health care, we would like to extend this call to seek any innovations in the use and applications of Open AI-based tools such as ChatGPT and ChatGPT-4 in the planning, implementation and/or evaluation of health behavior change interventions. To elaborate on this, ChatGPT and the newer version, GPT-4, are large language models which are designed to generate textual and graphic data from user-friendly commands such that researchers and students can generate information related to their research queries and possibly use some of it in their writings [1].

The use of digital communication has been in practice for the past couple of decades. However, over recent years, the use of artificial intelligence has become something of a new frontier in the fight to combat the rising incidence of death due to non-communicable diseases such as heart disease, diabetes, and obesity. Today, nearly 80% of American adults do not meet the guidelines for both aerobic and muscle-strengthening activity. At the same time, the prevalence of overweight or obese adults has reached 71.6% [2].

In a recently published scoping literature review addressing the role of machine learning in designing and implementing health promotion interventions for behavior change, multiple limitations such as burden on users for data input and the degree to which these could be personalized were noted [3]. Although this may be true, recent efforts have been made to support adolescent engagement in artificial intelligence-driven health behavior change interventions by designing and applying an ethically backed framework for accomplishing goals such as the measurement of adolescent engagement, the modeling of adolescent engagement, the optimization of current interventions and finally, the generation of novel interventions [4].

The purpose of this editorial is a timely assessment of existing practices in the use and application of open AI-based tools for health behavior assessment and/or change. In a recently conducted systematic literature review which addressed the role of chatbots based on AI for health behavior change, it was found that chatbots demonstrated high efficacy in several behaviors such as medication adherence, smoking cessation, substance misuse and overall healthy lifestyle maintenance. However, there were limitations noted, which were related to the generalizability of these effects across diverse populations and the need for robust randomized controlled trials in the future [5].

Another important aspect of the use of AI-driven technologies, for example, chatbots in health behavior change, deals with the ethics and ethical usage surrounding its application. Explicability, a specialized term which involves transparency and accountability, deals with explaining the workings of intelligent tools as well as understanding who is ethically and legally responsible for the interactions between chatbots and humans [6]. Thus, we see that there are additional components to keep in mind when designing and implementing intelligent technologies in health behavior interventions apart from the standard ethical framework of respect for persons, beneficence, non-maleficence, bias and justice [7]. In a recently conducted systematic review which tried to address ethics and artificial intelligence usage, particularly in the healthcare domain, some additional parameters to be considered were identified, such as privacy, dignity, sustainability, trust and cybersecurity, as overarching themes along with those mentioned earlier. So, clearly, there are many factors to be considered for designing health behavior interventions for behavior change, particularly in digital-based and eLearning environments for health education researchers and practitioners [8,9].

In conclusion, as the role of generative AI evolves, it would be interesting to see its integration in various phases of health behavior research. At the time of this writing, it is not clear what specific aspects of AI usage would be permissible in the research process, particularly in the conceptual development of health behavior interventions, the actual design of interventions and the on ground implementation of these for health behavior change. It is anticipated that AI will be playing a significant role in the design and implementation of health care and health behavior interventions in the near future, and hence, the valid question would be not whether to use and apply AI technology in health behavior change, but it would be how we can harness the potential of this technology in designing and implementing innovative digital-based health behavior interventions for a wide variety of behaviors.
